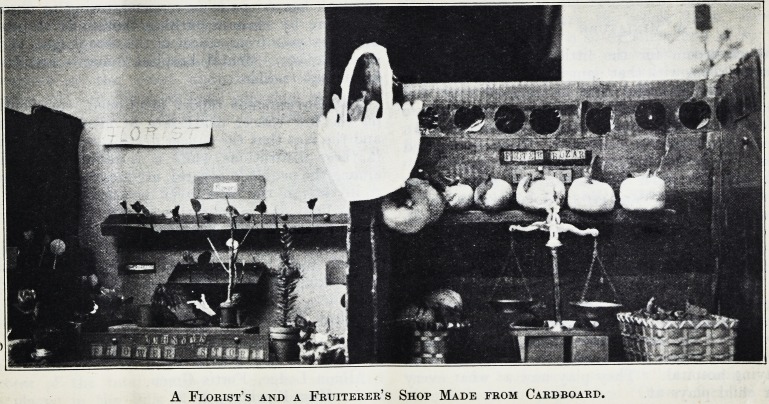# A Miniature Town from Hospital Waste

**Published:** 1924-12

**Authors:** 


### A MINIATURE TOWN FROM HOSPITAL WASTE.

### THE WORK OF CRIPPLED CHILDREN.

[From an American Correspondent.]

A remarkable example to schools and hospitals is to be found in a great New York hospital which is the only world that scores of little children have seen. Far from being institutionalised, these children, most of them crippled, are being taught everything that a normal child sees and knows. The ordinary home life, which some of them have never known, is brought directly to them by allowing them to construct tiny homes of their own and even stores, schools, churches—all that makes up knows and the only institution of which she sees the workings. Later on she, and her little boy friends as well, will " play shop " and gradually increase the extent of their imitative activities as they come into contact with more things of our everyday life. But what of the child who has seen little or nothing of a real home, a shop, or anything else of the world we know ? There are in hospitals to-day many little tots who have lived most of their lives within the walls of a hospital ward, which to

The Work of Crippled Children.

the existence of the average child. Once these youngsters, with only the white walls of the wards about them, could think only of " playing hospital," but now they imitate home life and do everything done by the little folk of the outside world. The miracle of it all is that these miniature homes and shops that the tots are making for themselves are all evolved from nothing but ordinary hospital waste. Old boxes, cans and paper become toy beds, chairs, lamps and fully furnished rooms. Not only are they helped thus in a social and educational way, but the work aids them to a speedier recovery and an earlier return to the real outside world.

### The Institutionalised Child.

Why is it that every little girl, as soon as she is old enough to play at all, likes to " play house " ? Simply because the house is the only world that she knows and the only institution of which she sees the workings.

them is the whole world of their experience. They have little idea of what constitutes household furniture, or what it is used for. They have, in fact, become institutionalised.

### Incurables at Play.

What, then, will be the nature of the play of these boys and girls when they come to the same imitative age that all children reach ? It would seem natural that they would " play at hospital," and this is actually what they do. Dolls were made ready for the operating table, or given medicine for various ills, but never were they dressed for parties or taken for walks. When one little girl asked another how her dolly was feeling, the grim answer often came back, " We can't save her leg ; it'll have to be cut off." This seemed to be far from a normal state of mind for a child, so one of the teachers assigned to educational work in the ward of these unfortunate little ones decided on a plan that she thought would both amuse her pupils and bring them into touch with the great outside world of which they knew so little from personal experience. They could not have real houses in which to become familiar with home life, but they could have miniature homes for toys. And they could have even more fun by building these themselves. For a long time there had been great piles of cardboard cartons, drug boxes, ether cans, bandage papers, and other materials generally classified as waste consigned to the hospital rubbish heap. For who would have thought that one of these huge packing cartons, such as those in which the large baking companies deliver bread, would make the framework for a wonderful house or shop or a church? And who could have believed that cardboard boxes might be transformed into ingenious little beds or ether cans into floor lamps? No one could have guessed that the coloured paper inside bandage containers and other cartons would make beautiful wall-paper.

A Bedroom Made by a Crippled Child.

### Cardboard Houses.

This is just what was done. The home was the first world that the children set about to create for themselves. Every child likes to have a little space somewhere that he can consider all his own—a favourite corner, a private spot behind a door or a screen, some place that will be entirely his own domain. Very much space could not be given to these youngsters in the crippled children's ward for their personal domains, and the miniature village became the way out. As soon as each child received his big cardboard box, he cut out windows or designated them by pasted-in slips of paper. Tiny curtains followed, and then the interior was transformed by the addition of wall-paper that was once the ordinary blue paper found about every bandage roll. The tiny room was already prepared for furnishing by the little hands of its owner. One youngster, while lying on a "prone board," his legs held down by weights, evolved the most remarkable sitting and dining rooms with some little co-operation from his friends. "Now you hold it, Mikey, and I'll cut it for you," went far to overcome all sorts of difficulties. The blue wall-paper became obsolete when Tommy, aged five, showed his talents as a painter. Tommy could never sit down because his brace hurt him, and he could stand up only for a little while at a time, but it was he who became "boss painter" of the community until he was transferred to another hospital, which he will probably never leave. In the teacher's own words, "If you want to see the Golden Rule lived, go into a children's ward in a hospital."

### "Occupational Therapy" for Children.

When the fitting up of the interiors of the toy houses became necessary, the little minds had to absorb new ideas about a world with which every child but them was familiar, and the tiny hands and fingers at the same time derived immense benefit from the pleasant work. Occupational therapy, it might have been called, but it was simply "play"

A Florist's and a Fruiterer's Shop Made from Cardboard.

to the teachers and pupils. The muscles which had been exercised so little took on new life when plying a needle, pasting a chair together, or papering a wall. A few minutes of this concentrated effort was as valuable as hours of massage. As to new knowledge, to the children who had spent their entire lives in the institution everything was new. But even to those who had come in after living in the world outside many things were unfamiliar, and many ideas had to be dropped. For instance, there was the tot who did not see why her house should not have a bed with two mattresses next to the kitchen stove, because "that's the way my mother's house is." Pianos, standing lamps and many pieces of bedroom furniture were unknown to children who had come to the hospital from poor homes.

### Ingenious Transformations.

Scrubbing boards were evolved from the corrugated paper around electric light bulbs, and carpet sweepers were born from old match boxes. A powder box, a pencil, and an ether can made a splendid floor lamp. An ordinary oblong pasteboard box could make an excellent doll's bed without any trouble at all, especially when neatly decorated with coloured paper. Pillows and coverings for the dollies were made by the youngsters themselves, and some of the latter bore the occupant's name. The children soon began to turn the empty bread boxes into other things besides rooms. Stores, churches, garages, schools, and even a hospital were soon to follow. When Tony, whose father runs a shop, entered the ward, his first effort was to produce a miniature of his father's shop. Then there was a day when everybody was making trains, a station and a booking office. In the same way a great number of automobiles designed by the children caused the making of a public garage. John, the son of an ironmonger, immediately introduced a shop like his father's into the growing community. When a coloured orchestra gave an entertainment for the children, one four-year-old tried to make himself a banjo, and a music shop with every variety of instrument was the outcome.

### A Miniature Town.

The classroom for the little ones, formerly an operating room with that name still engraved on the door, and the powerful light for surgical work still suspended from the ceiling above the space where the operating table once was, now resembles an ordinary kindergarten classroom. These rooms and stores, in a double line, more than half encircle the room, and the other sides are lined with various products of the boys and girls. A large round table in the middle permits a class to sit together and do their work with each other's help. Shop-made toys and doll's houses and furniture could have been provided, but the children would not have derived half so much pleasure as they do from making these things themselves and building a veritable town in miniature. What most firmly establishes the value of this novel work among the children is that since it began none among them has suggested "playing hospital." They play now at what every other child plays at.

## Figures and Tables

**Figure f1:**
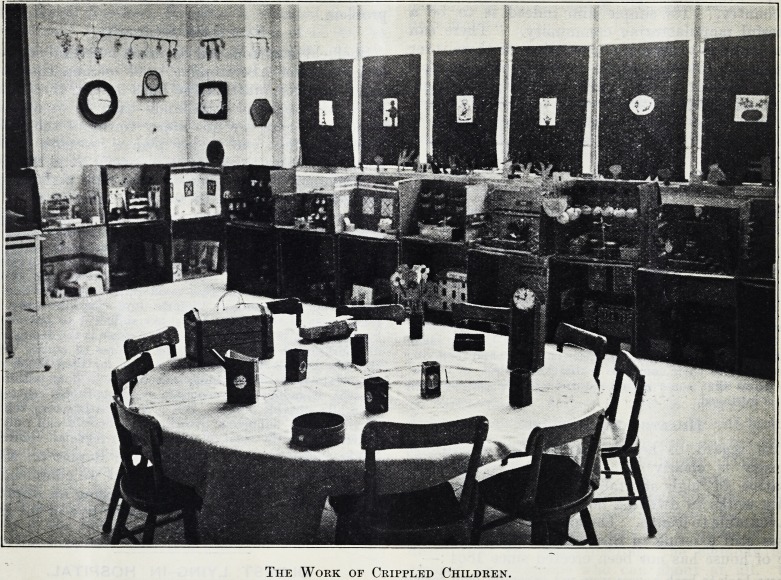


**Figure f2:**
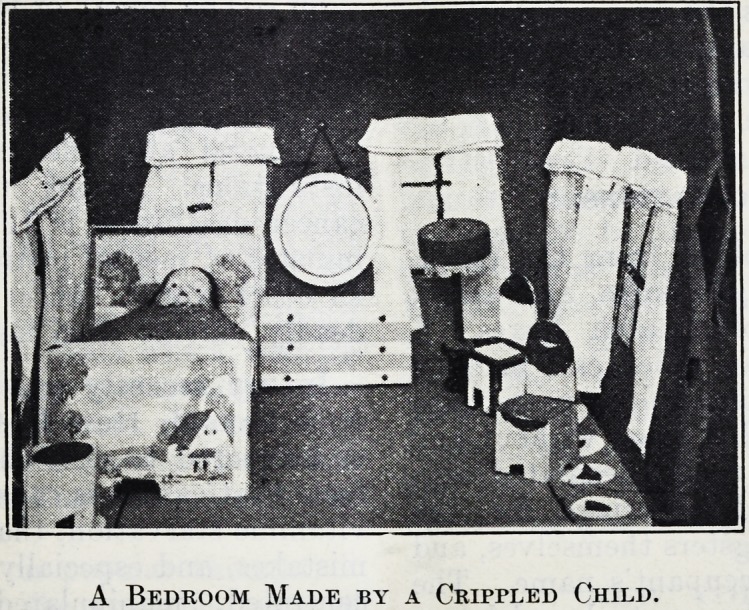


**Figure f3:**